# Human Breath Analysis May Support the Existence of Individual Metabolic Phenotypes

**DOI:** 10.1371/journal.pone.0059909

**Published:** 2013-04-03

**Authors:** Pablo Martinez-Lozano Sinues, Malcolm Kohler, Renato Zenobi

**Affiliations:** 1 ETH Zurich, Department of Chemistry and Applied Biosciences, Zurich, Switzerland; 2 Pulmonary Division, University Hospital Zurich, Zurich, Switzerland; 3 University of Zurich, Zurich Center for Integrative Human Physiology, Zurich, Switzerland; Gentofte University Hospital, Denmark

## Abstract

The metabolic phenotype varies widely due to external factors such as diet and gut microbiome composition, among others. Despite these temporal fluctuations, urine metabolite profiling studies have suggested that there are highly individual phenotypes that persist over extended periods of time. This hypothesis was tested by analyzing the exhaled breath of a group of subjects during nine days by mass spectrometry. Consistent with previous metabolomic studies based on urine, we conclude that individual signatures of breath composition exist. The confirmation of the existence of stable and specific breathprints may contribute to strengthen the inclusion of breath as a biofluid of choice in metabolomic studies. In addition, the fact that the method is rapid and totally non-invasive, yet individualized profiles can be tracked, makes it an appealing approach.

## Introduction

Personalized medicine aims to tailor medical treatment to the individual characteristics of each patient [1]. With the advent of high-throughput genome sequencing techniques, the concept of personalized health care is about to become a reality in the clinic [2]. However, the genome alone cannot account for factors like life style, interplay with gut microbiome [3] or circadian cycle [4]–[6]. For this reason, mapping of the metabolome and relating it to sub-populations or even individuals will be critical to fully achieve the concept of personalized healthcare [7]. However, precisely because the metabolome accounts for external factors, it is subject to intra-individual variations that need to be characterized. To address this issue, recent studies have investigated the question whether individual metabolic phenotypes are stable during extended periods of time [8], [9].

As other biofluids, breath contains relevant biochemical information, since it carries a large fraction of the most volatile metabolites [10]. Breath analysis is completely non-invasive, and therefore an attractive approach, which in principle is also suitable to monitor an individual's health status over extended periods of time. However, breath analysis has not yet been routinely used to complement the analysis of other biofluids in order to contribute to an individualized healthcare. This situation may start to be reversed by the assessment of the intra-individual variations of the composition of human breath; and ultimately examining whether or not individualized breathprints persist over the time. This has been the main goal of the present study.

## Materials and Methods

### Subjects

Eleven subjects [6 males/5 females; age 29.8±4.6 years (mean ± SD)] were included in this study. The subjects were ETH staff with a heterogeneous ethnic background (see Supplementary table S1 for details). During the period examined, the subjects did not change their routine life style. They came to our facilities during nine working days and their breath was analyzed during four time slots: 8 AM–11 AM; 11 AM–1 PM; 1 PM–3 PM and 3 PM–6 PM). Note that not all of them could attend every measurement; therefore, the total number of samples collected per subject ranged between 10 and 26 (average of 18). The order in which the subjects' breath was analyzed was randomized. To minimize confounding effects, the participants refrained from eating, drinking and brushing their teeth at least 30 minutes prior to the measurements. None of the subjects was a smoker and their routine dietary habits were kept constant. The Research Ethics Committee of ETH Zurich approved the study (EK 2012-N-25), and all subjects gave written informed consent to participate.

### Mass spectrometric analyses

The participants were asked to breathe through a heated Teflon tube (3 mm i.d.) connected to the curtain gas port of a quadrupole time-of-flight mass spectrometer (Q-TOF Ultima, Waters Inc.). The sampling tube was surrounded with a heating tape at 90 °C to prevent water condensation and to minimize losses onto the walls of exhaled compounds. Each time, a subject provided a full exhalation, while keeping the pressure through the sampling line at 20 mbar (typically +/− 2mbar;monitored by a digital manometer; this translated to a flow rate of 3.8 L/min). This process was performed in triplicate. The exhaled breath encountered an electrospray plume, where some compounds get ionized and subsequently are mass analyzed. This technique has been referred to as secondary electrospray ionization (SESI) [11]. The spray was formed with a home built source by infusing water (0.2% formic acid) at ∼100 nL/min through a PicoTip emitter (20 i.d; 360 o.d.) held at 2 kV (∼400 nA) above the sampling orifice. The electrospray tip was located 6 mm from the sampling cone and 1 mm off the symmetry axis.

### Data analysis

The three replicate mass spectra of each sample were averaged with MassLynx (Waters) and exported as txt files (m/z-intensity pairs). In this process, only the last few seconds (typically around 6 sec.) of each exhalation was considered, excluding from the analysis the first part of the exhalation which reflects mostly the dead volume in the upper respiratory tract. Further analysis was conducted with Matlab (R2011b, Mathworks Inc.). The original mass spectra were interpolated to 10,000 m/z values (56–400 Da, in steps of 0.0187 Da). The spectra were normalized by standardizing the area under the curve to the total median. After individually normalizing every signal, they were further scaled to adjust the overall maximum intensity to 100. Finally a 193×10,000 matrix was assembled.

First, we applied a Kruskal-Wallis test [12] to filter the most informative features regarding donor-specificity. The condition to retain an m/z value for further analysis was twofold: P<10^−3^ and in addition, at least one pair of measurements must be significantly different upon a further multicomparison test (Bonferroni corrected, 95% confidence level). These conditions were satisfied by 2,928 m/z signals. Further multivariate analysis to determine the existence of individual breathprints was pursued by combination of standard multivariate methods as previously described [8], [9]. Essentially, the dimensionality of the 193×2,928 matrix was further reduced by principal component analysis (PCA) and canonical analysis (CA). The PCA score sub-matrix (99.99% of explained variance) was subjected to MANOVA, which as performed by MATLAB computes CA, yielding 10 dimensions (all with P<10^−3^) maximizing the separation of the 11 subjects. The interpretation of the resulting discriminant functions to ascertain the contribution of the different mass spectral signals to the separation between subjects, was performed by standardizing the combined loading coefficients of PCA and CA as described previously [13], [14]. To visualize the contribution of the resulting 10 canonical dimensions to the discrimination of the subjects, they were used as input to compute hierarchical clustering (Euclidean distance).

Finally, the Kruskal-Wallis/PCA/CA model was cross-validated (train size = 182; test size = 11). The 11 test breath mass spectra were projected onto the subspace generated by the 182 training mass spectra and individually classified with a k-nearest neighbor classifier (k = 1; Euclidean distance). This process was repeated 500 times, shuffling in each iteration the test and training samples (Monte Carlo repartitions).

## Results and Discussion

**Figure 1:**
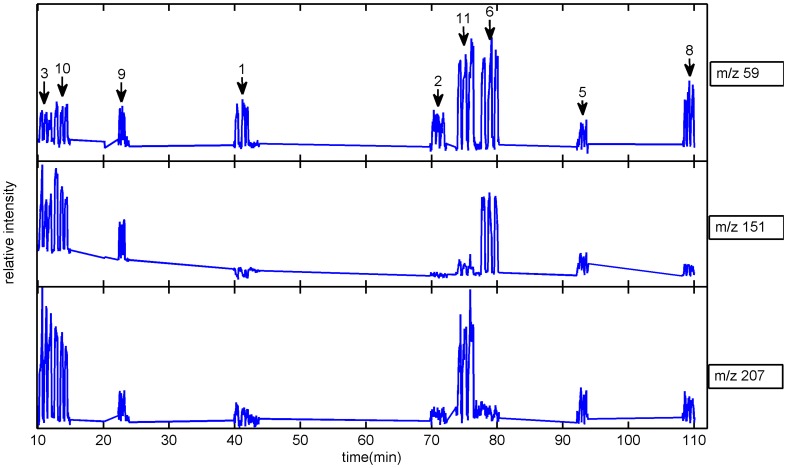
Real-time analysis allows for the rapid breathprinting of subjects. During the 1.5 hours experiment shown above, nine subjects breathed into the mass spectrometer. The subjects' label codes are indicated at the top of each of the three replicate measurements in the top trace. The three traces correspond to the ion intensity as a function of time for m/z 59, 151 and 207. Each subject breathed in triplicate, which is illustrated by the three steps of the signal above the background per subject. This snapshot already illustrates the high inter-subject breathprint variability.

SESI-MS allows for real-time breathprinting, producing rich mass spectrometric signatures [15]–[19]. This is illustrated in Figure 1, in which three examples of m/z values found in the breath of nine of the subjects are plotted. As noted above, not all the subjects could regularly attend every measurement. In the particular example shown in figure 1, subjects *4* and *7* were not present. Each trace corresponds to an ion's intensity as a function of time. During the 1.5 hours of the time trace shown, nine subjects breathed into the system (subject-label code indicated with arrows). The signal steps appearing above the background level result from a single exhalation, and it can be appreciated how the three replicate measurements per subject show comparable heights. Note that it is obvious already at this stage that each subject shows a distinct breathprint. For example, the ion at m/z 59 (acetone, based on a previous characterization [15]), is present in all subjects' breath, but in highest concentrations for subjects *6* and *11*. This high individual variability is consistent with previous measurements of breath acetone [20], [21]. However, figure 1 constitutes just a snapshot reflecting part of the exhaled breath composition at a given moment (morning measurement; 8 AM–11 AM). In the present study, we sought to assess the amplitude of the intra-individual variations reflected in fluctuations of exhaled breath composition over the course of several days, including intra-day measurements (i.e. morning and afternoon).

**Figure 2:**
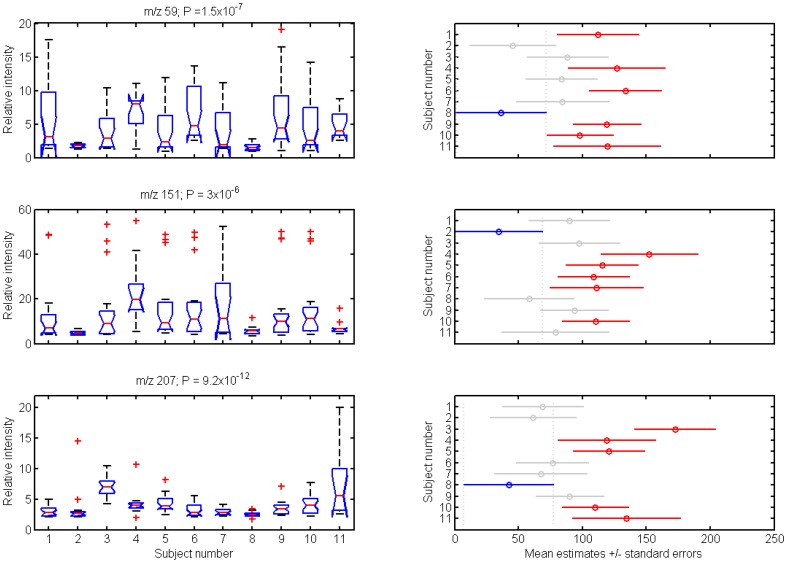
Temporal variability of the three compounds shown in **figure 1** over the nine days of measurements for the eleven subjects (left). The central mark of each box corresponds to the median, the edges of the box are the 25^th^ and 75^th^ percentiles, the whiskers extend to the most extreme data points not considered outliers, and outliers are plotted individually (beyond +/–2.7σ). The P values resulting from the Kruskal-Wallis test are quoted on top of the box-plots. Further multicomparison tests (right) showed that, with some exceptions, in most of the cases inter-subject variability was not significantly different (overlapping intervals).

This is illustrated in Figure 2 for the three representative ions of figure 1. The box plots provide an overview of the intra- and inter-subject variability during the whole period. For example, acetone (m/z 59) seems to have a greater intra-subject variability than m/z 207. This is also consistent with previous work [20], showing that inter- and intra-subject diurnal levels of breath acetone can be vary widely. Similarly, large variations of ethanol and acetaldehyde were found in a longitudinal study over a 6-month period [22]. We further assessed whether or not intra-subject variability were significantly different via a multicomparison (Bonferroni corrected). The results are summarized in the right hand side panels of figure 2, which show the computed mean ranks (95% confidence interval). Two means are significantly different if their intervals are disjoint, and are not significantly different if their intervals overlap. Thus, for example, subjects 2 and 8 show particularly low levels of these three compounds and are significantly different of several of the other subjects. The rest of the subjects show overlapping values, suggesting that further multivariate methods are needed to reveal the existence of highly donor-specific breathprints.

**Figure 3:**
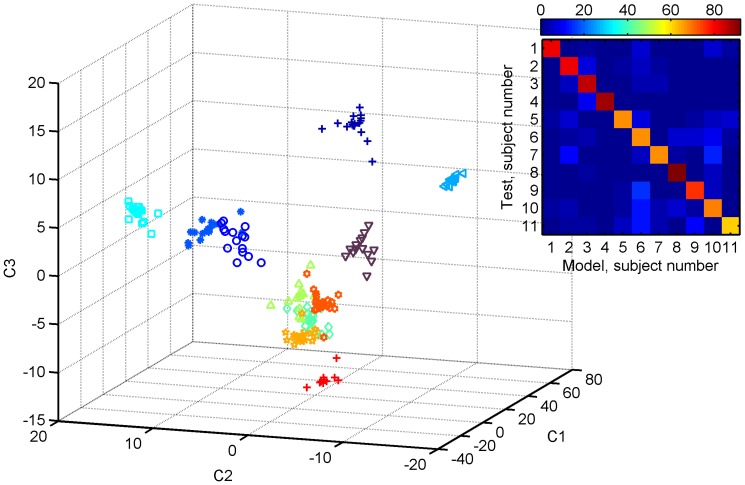
Projection of the 193 breath mass spectra onto the first three dimensions obtained by supervised Kruskal-Wallis/PCA/CA. Grouping according to breath donor (represented by different colors and symbols) becomes apparent. The inset displays the blind classification results upon a cross-validation (average of 500 Monte Carlo repartitions). The overall breath-mass spectrum to breath-donor recognition score was 76%.

This was done by PCA/CA analysis and the results are illustrated in figure 3, which shows the projection of the 193 individual mass spectra onto the first 3 PCA/CA dimensions. Clearly, each subject tends to occupy his/her own space, suggesting that each individual bears a unique breathprint, which is stable during at least eleven days (nine workdays + weekend). The relative contribution of the mass spectral signals to each of the 10 PCA/CA dimensions can be visualized in the loading plots shown in Supplementary Figure S1. The corresponding mean signal intensities and 95% confidence intervals of the most diagnostic signals are listed in Supplementary table S2.

A sharper separation could be obtained by accounting for the 10 canonical dimensions through hierarchical cluster analysis (Supplementary Figure S2), whereby each of the breath mass spectra clustered according to donor. Finally, the Kruskal-Wallis/PCA/CA model was cross validated (n = 11 for test; n = 182 for model). The inset of Figure 3 summarizes the classification rate results upon 500 Monte Carlo repartitions. The reddish diagonal indicates that most of the times the breath mass spectra presented to the model were correctly assigned to the donor. More precisely, the overall recognition score was 76%, and the individual correct classification rate ranged from 62% (subject 11) to 92% (subject 8). It is interesting to note that, in the former case only 10 samples were available for this subject. Therefore, at best 9 breath samples were available in the model during the k-fold cross validation. This is in line with the learning curves found in Figure 4a of Ref. [8], were the probability of correct classification with 5 NMR spectra in model set was around 58% (median of the box plot), 75% for 10 spectra and reached 100% with some 30 spectra. Note also that in this study we have included samples from different time slots. Therefore, since diurnal variations in the composition of exhaled breath are believed to occur [23], this may add further heterogeneity to the “characteristic individual fingerprint”, thus calling for larger model datasets to cover this intra-day variability.

Overall, this data is consistent with previous work based on urine showing that small biomolecules released into human biofluids can be analyzed, and with support of sophisticated statistical analysis, individual signatures (metabotypes) can be revealed [8], [9]. Moreover, we have included data collected during different times of the day. It is known that part of the metabolome fluctuates as a result of biological clocks like for example the circadian cycle [4]. We found that, despite this potential source of within day variation, the individual signatures were yet identifiable. These results are encouraging; however, one limitation of this study is the lack of positive identification of most of the breath components. For this reason, it is difficult to determine to what extent exogenous compounds (e.g. due to exposure to chemicals in a laboratory) may ultimately contribute to produce individualized exhaled fingerprints. Ongoing investigations with a higher resolving power instrument than the one used in this study are expected to provide structural elucidation of the most significant exhaled compounds and therefore ultimately determine to what extent endogenous metabolites contribute to the individual breathprints identified in this study. Despite this noted limitation,,this study suggests that breath may be incorporated into metabolomic studies as a valuable additional source of information, complementary to the analysis of other biofluids like plasma or urine. In addition, even though its analysis in real-time does not provide sets of information as rich as traditional off-line techniques, it suffices to track breathprints with individualized precision. Given that breath analysis is simple, non-invasive and rapid, we envisage its application as an individualized screening tool; whereby the deviation of one's breath signature beyond the natural “daily noise”, may trigger further examinations to determine the reasons.

## Conclusions

Based on real-time mass spectrometric analysis of exhaled breath of a group of subjects during nine days (up to four measurements per day), we conclude that: i) the intra- and inter-subject variability for some compounds detected in breath can vary relatively widely; ii) despite this intra-individual variations, stable “core” breathprints, which are highly donor-specific could be identified; iii) overall, this suggests that breath analysis may contribute, together with other techniques, towards a future individualized-oriented healthcare.

## Supporting Information

Figure S1 Relative signal contributions to each of the 10 PCA/CA dimensions found to maximize the separation of the breathprints of 11 subjects. Positive (negative) values indicate relatively increased (decreased) intensities in subjects with positive values in the corresponding dimension (i.e. reddish color in clustergram; Figure S1) as compared to those with negative values (i.e. greenish in clustergram; Figure S1).
(TIF)

Figure S2 Clustergram resulting from hierarchical cluster analysis for the 10 Kruskal-Wallis/PCA/CA dimensions (rows). The dendrogram on the top displays 11 clusters corresponding each to one individual (color coded; M: Male; F: Female). The heatmap provides an overview of the relative contribution of each of the 10 dimensions to each individual cluster (red: positive values; black: values close to zero; green: negative values). Intra-subject distances are smaller than inter-subject ones, suggesting the existence of individual breath phenotypes.
(TIF)

Table S1 Characteristics of this study subjects.
(DOCX)

Table S2 Mean signal intensities and 95% confidence interval for the mean (in parentheses) for the most diagnostic ions (i.e. higher absolute global loading coefficients). The p-values reported for each m/z were computed according to a Kruskal-Wallis test.
(DOCX)
