# Supplementary figures and images for: Human Breath Analysis May Support the Existence of Individual Metabolic Phenotypes
Source: PLoS One. 2013 Apr 3;8(4):e59909. doi: 10.1371/journal.pone.0059909 (PMC3616042; doi:10.1371/journal.pone.0059909)

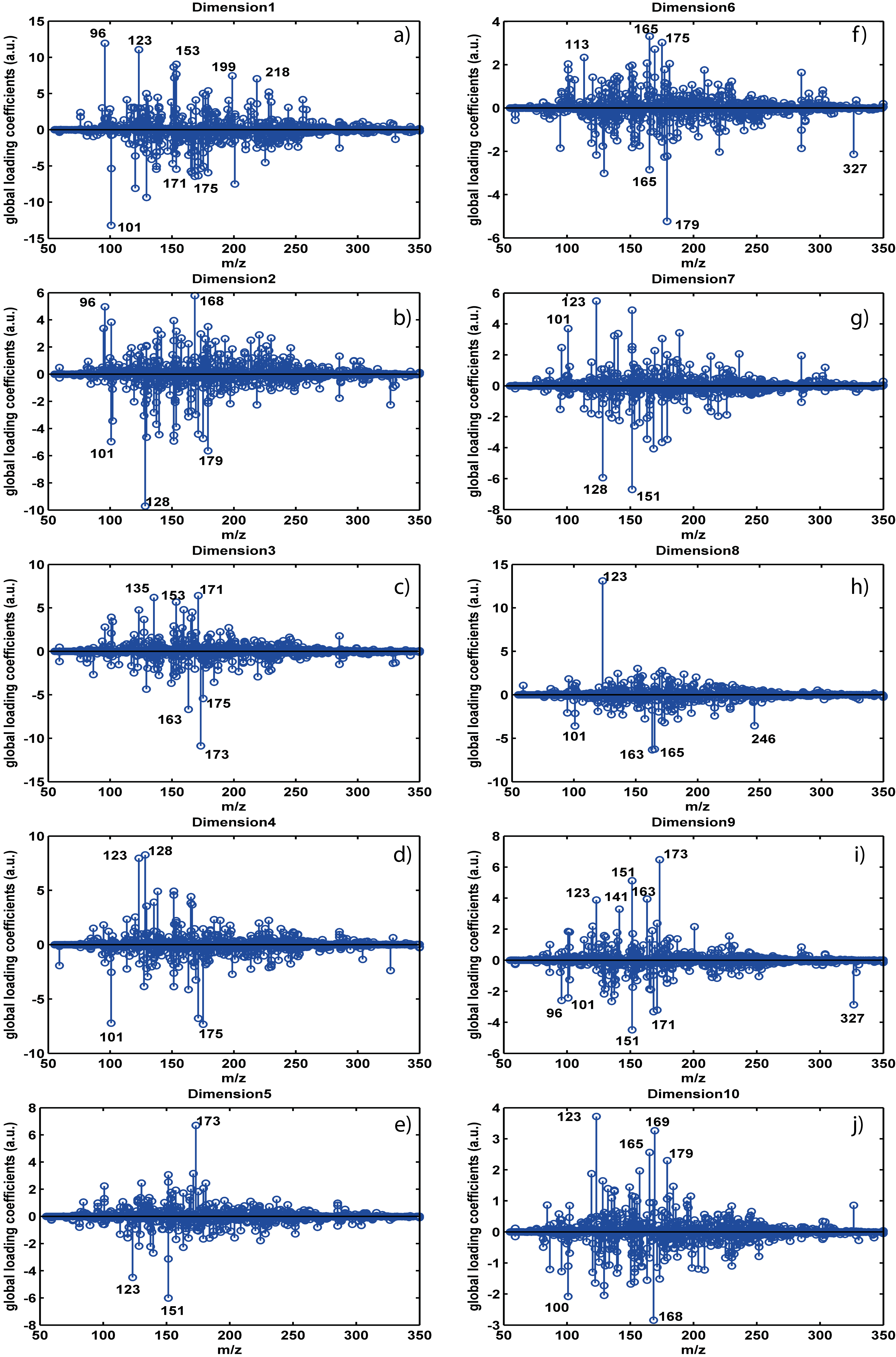

Supplement: Figure S1 — Relative signal contributions to each of the 10 PCA/CA dimensions found to maximize the separation of the breathprints of 11 subjects. Positive (negative) values indicate relatively increased (decreased) intensities in subjects with positive values in the corresponding dimension (i.e. reddish color in clustergram; Figure S1) as compared to those with negative values (i.e. greenish in clustergram; Figure S1). (TIF) [file pone.0059909.s001.tif]

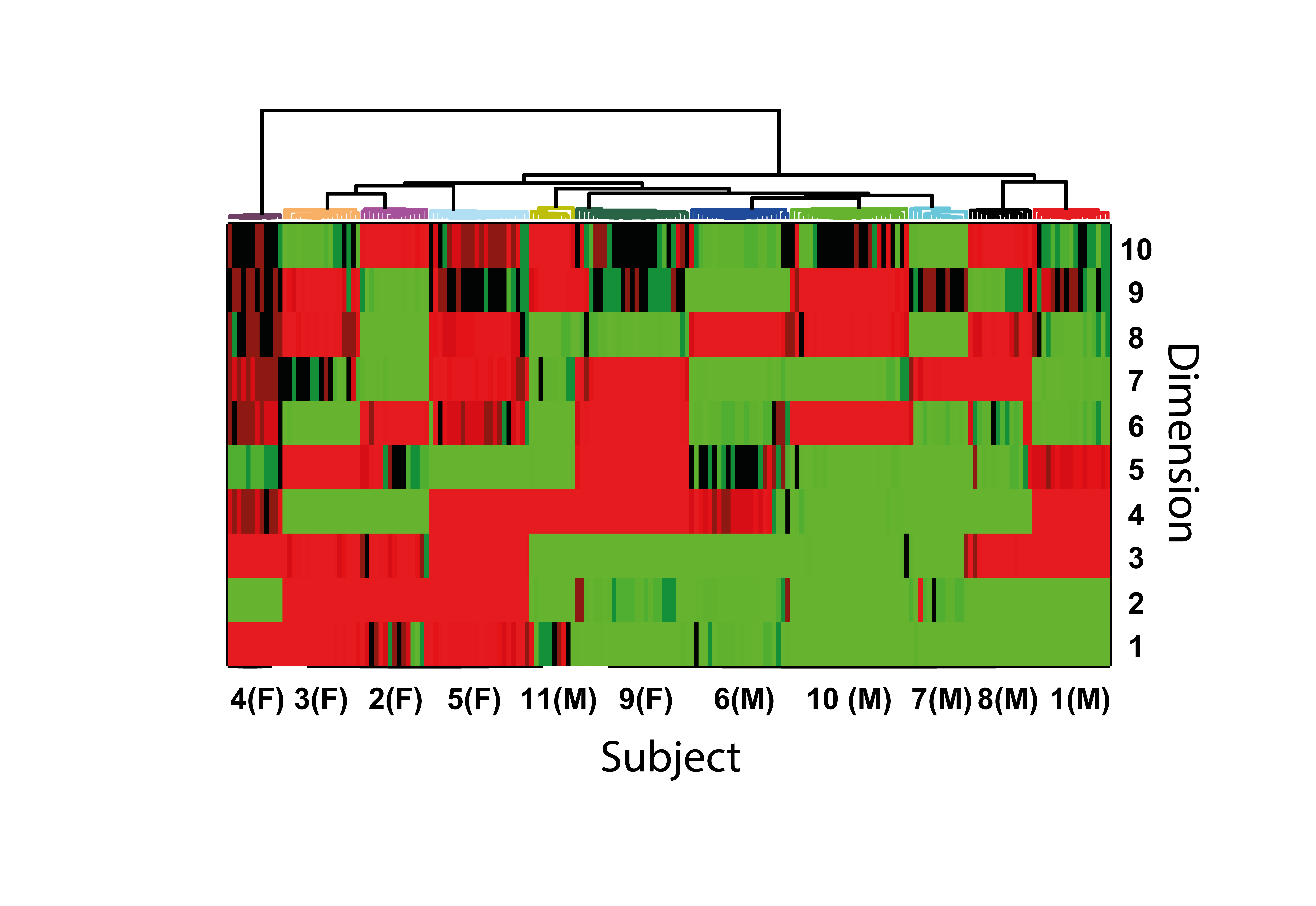

Supplement: Figure S2 — Clustergram resulting from hierarchical cluster analysis for the 10 Kruskal-Wallis/PCA/CA dimensions (rows). The dendrogram on the top displays 11 clusters corresponding each to one individual (color coded; M: Male; F: Female). The heatmap provides an overview of the relative contribution of each of the 10 dimensions to each individual cluster (red: positive values; black: values close to zero; green: negative values). Intra-subject distances are smaller than inter-subject ones, suggesting the existence of individual breath phenotypes. (TIF) [file pone.0059909.s002.tif]
