# Supplementary material for: Human Breath Analysis May Support the Existence of Individual Metabolic Phenotypes
Source: PLoS One. 2013 Apr 3;8(4):e59909. doi: 10.1371/journal.pone.0059909 (PMC3616042; doi:10.1371/journal.pone.0059909)
Supplement: Table S1 — Characteristics of this study subjects. (DOCX) [file pone.0059909.s003.docx]

**Supporting Information table 1**

| **Subject (number of breath samples)** | **Gender** | **Age** | **Ethnical background** |
| --- | --- | --- | --- |
| 1 (n=17) | M | 35 | Latin America |
| 2 (n=15) | F | 28 | Asia |
| 3 (n=17) | F | 26 | Western Europe |
| 4 (n=12) | F | 37 | Western Europe |
| 5 (n=22) | F | 26 | Asia |
| 6 (n=22) | M | 26 | Eastern Europe |
| 7 (n=13) | M | 26 | Western Europe |
| 8 (n=14) | M | 34 | Western Europe |
| 9 (n=25) | F | 28 | Asia |
| 10 (n=26) | M | 36 | Western Europe |
| 11 (n=10) | M | 26 | Western Europe/Asia |

**Table S1**. Characteristics of this study subjects.
