# Supplementary material for: Human Breath Analysis May Support the Existence of Individual Metabolic Phenotypes
Source: PLoS One. 2013 Apr 3;8(4):e59909. doi: 10.1371/journal.pone.0059909 (PMC3616042; doi:10.1371/journal.pone.0059909)
Supplement: Table S2 — Mean signal intensities and 95% confidence interval for the mean (in parentheses) for the most diagnostic ions (i.e. higher absolute global loading coefficients). The p-values reported for each m/z were computed according to a Kruskal-Wallis test. (DOCX) [file pone.0059909.s004.docx]

**Supporting Information table 2**

| **m/z [p-value]**  **subject** | **59 [1E-7]** | **96 [5E-7]** | **101 [8E-4]** | **113 [1E-4]** | **123 [1E-4]** | **128 [7E-4]** |
| --- | --- | --- | --- | --- | --- | --- |
| 1 | 6.2 (3.6-8.7) | 12.4 (8.1-16.8) | 21.8 (15.9-27.6) | 23.3 (15.4-31.1) | 50.8 (39-62.6) | 35.6 (27.8-43.3) |
| 2 | 1.7 (1.6-1.9) | 3.3 (2.9-3.7) | 12.3 (10.8-13.7) | 42.2 (35.2-49.1) | 30.9 (28.6-33.2) | 21.2 (19.8-22.7) |
| 3 | 3.9 (2.4-5.5) | 9.1 (6-12.1) | 20.4 (14.3-26.5) | 32.5 (23.4-41.6) | 42.7 (30.7-54.7) | 31.8 (23.2-40.4) |
| 4 | 6.7 (4.8-8.6) | 15.2 (11.1-19.2) | 30.4 (23-37.8) | 11 (8.4-13.6) | 66.7 (53.1-80.3) | 50 (38.7-61.4) |
| 5 | 4 (2.6-5.4) | 8.1 (5.3-10.8) | 21.6 (15.8-27.3) | 28.2 (20.7-35.7) | 49.1 (38.8-59.5) | 33 (25.4-40.6) |
| 6 | 6.7 (4.9-8.5) | 12.1 (8.6-15.5) | 21.6 (16.6-26.7) | 29.3 (21-37.6) | 48.4 (37-59.7) | 35.3 (27.6-42.9) |
| 7 | 4.3 (2-6.6) | 9.3 (4.4-14.3) | 20.4 (12.3-28.4) | 30.1 (17.5-42.7) | 47.9 (33-62.8) | 34.9 (22.9-47) |
| 8 | 1.6 (1.3-1.9) | 4 (3.3-4.7) | 11.4 (10-12.7) | 41.5 (36.4-46.6) | 29 (27.3-30.7) | 22.3 (21-23.7) |
| 9 | 6.6 (4.4-8.9) | 11.8 (8-15.7) | 21.1 (16-26.3) | 25.6 (19-32.2) | 46.2 (36.7-55.6) | 32.9 (26.3-39.5) |
| 10 | 4.9 (3.3-6.6) | 10.7 (7.1-14.4) | 20.9 (15.8-26.1) | 28.9 (21.3-36.5) | 44.3 (35.1-53.4) | 33.8 (27.2-40.5) |
| 11 | 4.8 (3.4-6.2) | 6.2 (4.7-7.8) | 11.6 (10.2-12.9) | 36.5 (30.3-42.7) | 28.5 (24.2-32.9) | 20.1 (18.3-21.8) |

| **135 [5E-11]** | **141 [7E-4]** | **151 [7E-7]** | **153 [1E-4]** | **163 [6E-8]** | **165 [6E-4]** | **168 [8E-4]** | **169 [6E-5]** | **171 [2E-5]** |
| --- | --- | --- | --- | --- | --- | --- | --- | --- |
| 19.7 (17.4-21.9) | 20.2 (17.8-22.6) | 18.5 (5.6-31.3) | 15.7 (13.4-18.1) | 20.6 (15.9-25.3) | 24.9 (19.6-30.3) | 7.9 (1.1-14.6) | 11.5 (7.7-15.3) | 19.7 (15.9-23.5) |
| 11.9 (10.1-13.8) | 13.6 (12.7-14.5) | 6.2 (5.7-6.7) | 11 (9.5-12.4) | 10.9 (9.6-12.3) | 18.4 (16-20.8) | 1.1 (1-1.2) | 5 (4.7-5.2) | 12.7 (11.1-14.4) |
| 14.6 (13.1-16) | 16.4 (14.5-18.4) | 23.6 (8.8-38.3) | 11.2 (9.8-12.6) | 15 (11.8-18.3) | 20.6 (16-25.1) | 8.8 (1.4-16.2) | 9.7 (6.3-13) | 16 (13.2-18.9) |
| 17.4 (15.7-19.2) | 20.6 (17.7-23.5) | 51.8 (30-73.7) | 13.8 (12-15.5) | 22.9 (19.6-26.2) | 29.4 (24.8-34) | 15.8 (5.8-25.9) | 18.3 (13.6-22.9) | 20.7 (17.8-23.6) |
| 13.7 (12.2-15.3) | 17 (15.2-18.8) | 23.1 (12.4-33.8) | 13.2 (11.5-14.9) | 15.7 (12.7-18.7) | 23.9 (19.6-28.2) | 8 (2.6-13.5) | 11 (7.7-14.2) | 17.2 (14.3-20.1) |
| 21.7 (18.6-24.8) | 17.9 (15.8-20.1) | 34.8 (21.7-47.9) | 12.8 (11.1-14.5) | 20.8 (16.8-24.8) | 23.8 (19-28.6) | 7.9 (2.2-13.6) | 10.8 (7.5-14.2) | 17.6 (14.4-20.8) |
| 16.7 (14.4-19) | 17.2 (13.8-20.5) | 31.8 (13.1-50.5) | 12.8 (10.5-15) | 17.7 (12.9-22.5) | 22.6 (17-28.2) | 11.6 (1-22.2) | 10.3 (6-14.6) | 16.7 (12.8-20.7) |
| 13.2 (11.2-15.2) | 14.8 (13.1-16.5) | 7.4 (6.1-8.7) | 9.6 (9.1-10.2) | 10.3 (9.7-10.9) | 14.5 (13.8-15.3) | 1.6 (1.2-2) | 5 (4.6-5.3) | 11 (10.7-11.3) |
| 19.5 (17.1-21.8) | 17.8 (16-19.6) | 20 (10.5-29.5) | 14 (12-16) | 20 (16.2-23.7) | 23.1 (19.1-27) | 6.8 (2.1-11.5) | 10.8 (7.8-13.7) | 18.4 (15-21.8) |
| 17.5 (15.3-19.7) | 17.5 (15.6-19.5) | 29.4 (18.8-39.9) | 11.9 (10.7-13.2) | 17.4 (13.9-20.9) | 22.1 (18.3-25.8) | 7.5 (2.3-12.8) | 10.3 (7.2-13.4) | 16.2 (13.6-18.8) |
| 27.4 (20.1-34.7) | 15.7 (14.3-17) | 16.7 (3.5-30) | 10.9 (8.5-13.3) | 17.1 (14.6-19.6) | 15.9 (14.2-17.6) | 1.3 (1.1-1.5) | 5 (4.5-5.5) | 13.9 (11.3-16.5) |

| **173 [7E-4]** | **175 [2E-5]** | **179 [5E-4]** | **199 [8E-5]** | **218 [2E-8]** | **246 [3E-4]** | **285 [8E-20]** | **327 [1E-3]** |
| --- | --- | --- | --- | --- | --- | --- | --- |
| 16.8 (8.5-25.1) | 25.2 (18.8-31.6) | 17.7 (13.6-21.8) | 15.3 (12.6-18) | 6 (4.5-7.6) | 12.6 (10.2-15) | 1.7 (1.4-2) | 10 (7.9-12.2) |
| 5.9 (5.4-6.4) | 14.7 (12.9-16.5) | 9.9 (9.2-10.7) | 15.1 (10.6-19.6) | 3.2 (2.9-3.4) | 8.4 (8-8.7) | 1.4 (1.1-1.7) | 6.9 (6.4-7.4) |
| 17.6 (7.5-27.6) | 18.3 (13.7-23) | 13.6 (10.3-16.9) | 13.6 (12.2-15) | 5.7 (4.3-7.1) | 11.1 (8.7-13.5) | 2.3 (1.9-2.7) | 9.2 (7.3-11.1) |
| 30.5 (16.5-44.6) | 26.5 (21.6-31.4) | 19.7 (16.2-23.2) | 14.4 (9.6-19.3) | 9.9 (8.1-11.7) | 16.1 (13.1-19.2) | 2.1 (1.7-2.5) | 12.9 (10.1-15.7) |
| 17.2 (9.6-24.8) | 22.4 (17.9-26.9) | 15.1 (12.2-18) | 13.3 (10.8-15.9) | 7.1 (5.1-9) | 12.5 (10.1-14.9) | 5.6 (2.3-9) | 10 (8.2-11.7) |
| 16.8 (9.5-24.1) | 21.2 (15.7-26.7) | 16 (12.4-19.6) | 16 (13.6-18.3) | 5.8 (4.5-7.2) | 12.2 (9.9-14.5) | 1.6 (1.3-1.9) | 10.1 (8.1-12) |
| 18.9 (5.7-32.1) | 21.3 (14.5-28) | 15.5 (10.9-20.2) | 16.6 (11.1-22.1) | 5.9 (4-7.7) | 11.8 (8.6-15) | 1.7 (1.3-2.1) | 9.2 (6.6-11.7) |
| 5.9 (5.3-6.4) | 12.8 (12.1-13.5) | 9.9 (9.4-10.4) | 12.7 (10.7-14.7) | 3.4 (3.1-3.8) | 7.8 (7.4-8.3) | 1.1 (1-1.2) | 6.2 (5.6-6.9) |
| 16.2 (9.3-23) | 22 (16.4-27.6) | 16 (12.4-19.6) | 15.9 (13.7-18.1) | 6.8 (5.4-8.2) | 12.1 (10-14.2) | 12.8 (8.7-16.9) | 9.8 (8.2-11.4) |
| 15.6 (9.3-21.9) | 19.2 (15.3-23.1) | 14.4 (11.5-17.3) | 13.8 (12.5-15.1) | 5.2 (4.1-6.3) | 11.3 (9.4-13.3) | 1.5 (1.3-1.7) | 9.1 (7.5-10.7) |
| 6.2 (5.6-6.8) | 13.3 (10.6-16) | 11.2 (9.6-12.9) | 24.1 (17.2-31.1) | 3.8 (3.4-4.3) | 8.8 (8.2-9.4) | 1.5 (0.9-2) | 6.5 (5.6-7.3) |

**Table S2**. Mean signal intensities and 95% confidence interval for the mean (in parentheses) for the most diagnostic ions (i.e. higher absolute global loading coefficients). The p-values reported for each m/z were computed according to a Kruskal-Wallis test.
